# Genome Characteristics of Vardy, a Cluster DJ Actinobacteriophage Isolated on Gordonia rubripertincta in Western North Carolina

**DOI:** 10.1128/mra.00704-22

**Published:** 2022-10-18

**Authors:** Montana Henson, Matteo Fratarcangeli, Serim Park, Ashley Minot, Maria D. Gainey

**Affiliations:** a Department of Biology, Western Carolina University, Cullowhee, North Carolina, USA; b Department of Chemistry and Physics, Western Carolina University, Cullowhee, North Carolina, USA; Queens College CUNY

## Abstract

Phage Vardy is a lytic siphovirus isolated from creek soil in Cullowhee, NC, using Gordonia rubripertincta NRRL B-16540. Vardy’s 60,144-bp genome contains 90 predicted genes and five copies of a 50-bp motif that may regulate gene expression. Based on gene content similarity, Vardy is assigned to cluster DJ.

## ANNOUNCEMENT

As the most abundant and genetically diverse biologic entities on Earth (1), bacteriophages are an invaluable resource for understanding basic biologic and evolutionary processes. Here, we report on Vardy, a bacteriophage isolated from soil collected alongside a creek in Cullowhee, NC (35.310278 N, 83.187222 W), using a standard enrichment protocol (https://seaphagesphagediscoveryguide.helpdocsonline.com/home). Briefly, a Ziploc bag was submerged into Cullowhee Creek and a soil sample was collected from the top 2 in. of the river bed. The soil sample was added to peptone-yeast-calcium (PYCa) medium containing Gordonia rubripertincta NRRL B-16540. The sample was incubated with shaking for 48 h at 30°C and filtered (0.22-μm pore), and the filtrate was plated in top agar with Gordonia rubripertincta. Three rounds of plaque purification were performed before a high-titer lysate was prepared (https://seaphagesphagediscoveryguide.helpdocsonline.com/home). Double-stranded DNA was purified using the Promega Wizard DNA cleanup kit, prepared for sequencing using the NEB Ultra II kit, and sequenced on an Illumina MiSeq (v3 reagents) to yield 511,940 150-bp single-end reads that constituted 1,202-fold coverage. Reads were assembled using Newbler v.2.9 and checked for accuracy and genomic termini using Consed v.29 (2). Default parameters were used for all software referenced in this article unless otherwise specified.

The genome is 60,144 bp with defined ends containing 3′ overhangs (CGCCGCTCT). The GC content is 51.2%—much lower than the 67 to 67.5% GC content of Gordonia rubripertincta strains reported in GenBank. Based on gene content similarity, Vardy was assigned to cluster DJ (3). Cluster DJ phages have siphoviral morphologies and similar GC content to Vardy, including phage Runhaar (GenBank accession no. MZ005683), which has the highest shared nucleotide identity (98.54%; 98% coverage) and was isolated on Gordonia terrae 3612 (4).

Glimmer v.3.02 (5) and GeneMark v.2.5p (6), embedded within DNA Master v.5.23.6 (http://cobamide2.bio.pitt.edu), were used to generate an automated annotation of the genome. Translational starts were then refined using PECAAN v.20211202 (https://discover.kbrinsgd.org/), Starterator v.459 (http://phages.wustl.edu/starterator/), and Phamerator Actino_Draft v.459 (7). Functions were assigned to open reading frames using top hits from HHpred (8), blastp (9) searches against the NCBI nonredundant and actinobacteriophage databases (4), tRNAscanSE v2.0 (10), and ARAGORN v1.1 (11). Ninety protein-coding genes, all transcribed in the forward direction, were predicted. No tRNAs were identified. Twenty-two genes were assigned functions, while 5 (genes 4, 35, 62, 68, and 75) contained no homologues among actinobacteriophages. The structural genes occur in the left arm beginning at gene 12 (terminase) and are preceded by genes coding for proteins of unknown function, an HNH endonuclease, lysin A, and a holin. The right arm of the genome contains DNA metabolism genes, including a DNA primase/polymerase gene (gene 60). No evidence of any genes coding for integrase or DNA partitioning proteins was found.

**FIG 1:**
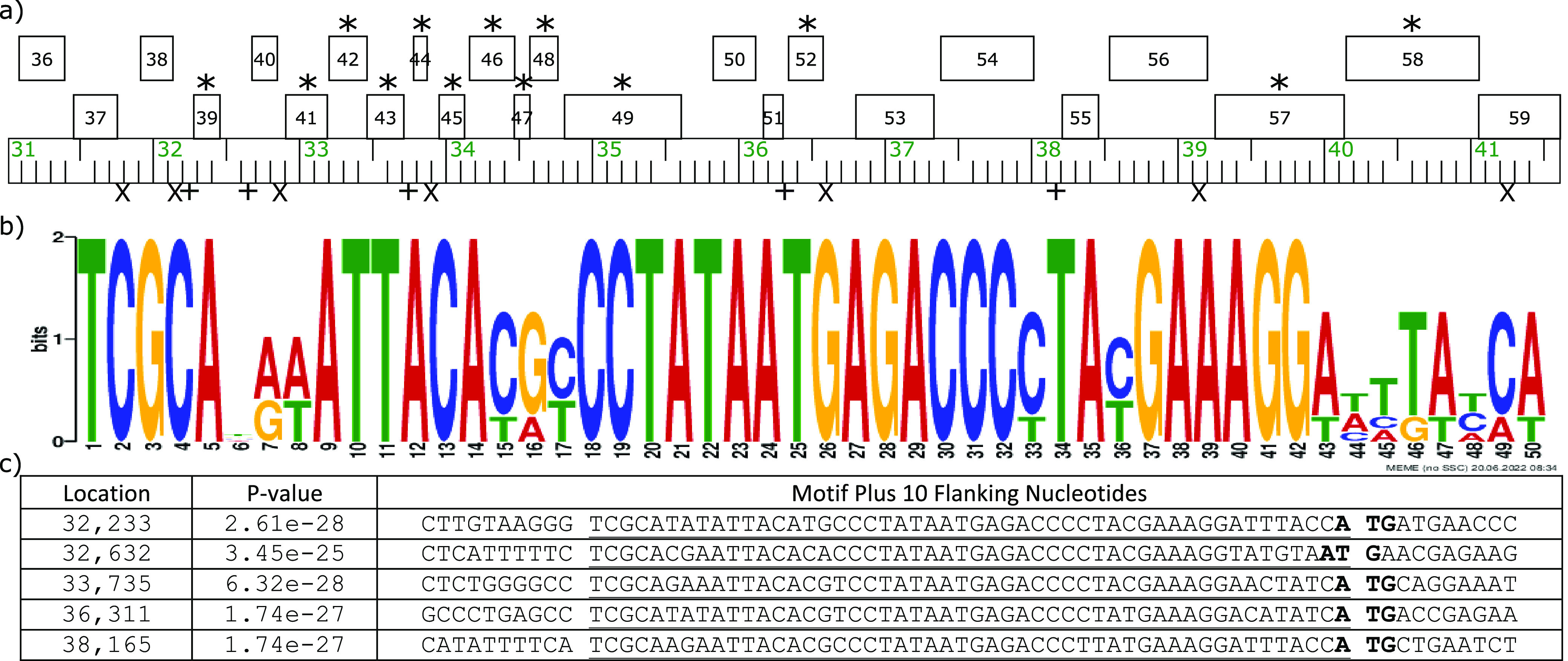
Genomic features of the region containing the 50-bp motif. Panel a shows a representation of the genome from genes 36 to 59, generated using Phamerator. Genes are represented with numbered boxes, and those predicted to contain a transmembrane or signal sequences are denoted with “*.” The locations of rho-dependent terminator sequences and the 50-bp repeats are represented with “X” and “+” symbols, respectively. The ruler shows the genome location in kilobase pairs. Panel b depicts the 50-bp motif logo generated by MEME analysis. The start location, *P* value, and sequence for each occurrence of the 50-bp motif, including 10 nucleotides flanking the motif, are shown in panel c. The predicted translational start site of the gene located downstream of each motif is in boldface, while the motif itself is underlined.

A MEME Suite 5.4.1 motif discovery search (12) revealed 5 occurrences of an intergenic 50-bp sequence in the middle region of the genome from genes 39 to 55 (Fig. 1). The motif contains a conserved TATAAT −10 sigma^70^ promoter sequence and an adenine at position 49 or 50 that likely serves as a translational start site (Fig. 1). Softberry FindTerm (13) and TOPCONS (13) analyses of this region predicted 6 positive-sense rho-dependent terminators and 13 genes that may contain a transmembrane or signal sequence motif, respectively (Fig. 1a).

### Data availability.

Vardy is available in GenBank under accession no. ON970622 and the Sequence Read Archive (SRA) under accession no. SRX14485101.
